# Injury as a Factor in the Production of New Growths of Bone

**Published:** 1894-01-06

**Authors:** A. H. Tubby

**Affiliations:** Surgeon to Out-patients, Evelina Hospital for Sick Children; Assistant Surgeon, National Orthopædic Hospital; Senior Demonstrator of Physiology, Guy's Hospital


### INJURY AS A FACTOR IN THE PRODUCTION OF NEW GROWTHS OF BONE.

By A. H. Tubby M.S.Lond., F.R.C.S.Eng., Surgeon to Out-patients, Evelina Hospital for Sick Children; Assistant Surgeon, National Orthopædic Hospital; Senior Demonstrator of Physiology, Guy's Hospital.

In this article I propose to show that in bony structures injury and new growth stand to one another in the relation of cause and effect.

The new growths of bone are of two varieties, innocent and malignant. The innocent growths assume either the form of cartilage or true bone, and are styled enchondroma or exostosis. We know that repeated irritation will produce what are known as spurious exostoses. An interesting example is found in the Adductor Magnus tendon in those who ride much. The tendon undergoes ossification, and a bony outgrowth ensues.

From many examples I select but one to give point to my proposition that these innocent growths follow injury. It is as follows: A boy, aged 14, three years previously received a blow from a cricket-ball on the inner side of the leg just above the knee. He experienced no inconvenience for twelve months but then began to have pain on walking. On flexing the knee he noticed a swelling. A month later, after a long walk, the swelling increased rapidly, and an operation was performed. There was found to be an elongated bony growth attached by a narrow base to the internal condyle of the femur, and not connected with the Adductor Magnus. On microscopical examination the growth showed an interior structure of soft and cancellous bone, and was covered with cartilage. In this connection it might be noted that injury of the soft parts are occasionally followed by formation of true fatty tumours or lipomata. Such tumours, exostoses and lipomata, are essentially of a benign nature; once removed they do not recur.

Far different from the foregoing is another variety of tumours that occur in bone after injury, viz., sarcomata. In 1885, at the Medical Society of London, Mr. Pearce Gould brought forward three cases of sarcoma of the bones following injury. The first of these was a girl, aged sixteen, who three months previously struck her forearm. She then noticed a swelling at the upper end of the radius, which enlarged rapidly till it involved the upper third of the bone. On operation it was found to be a giant-celled sarcoma. The second case occurred in a woman, aged twenty-six, who had struck her thigh three months previously. In two months' time, a swelling was apparent which steadily increased, so that when seen the whole bone was involved, and the tumour had attained a large size. Amputation was performed, and the tumour was found to be a sarcoma involving the whole thickness of the bone and the medullary canal. A third case occurred in an old man. Mr. McNamara states that 50 per cent. at least of the cases of sarcoma in bone are attributable to blows or injuries.

Bland Sutton remarks that in animals sarcomata are detected with the greatest frequency in the parts exposed to injury—in fishes, the tails and fins; in frogs, the limbs; in birds, the neck and prominent parts of the wings; in horses, sarcomata followed blows on the jaws. I might allude to many specimens of sarcoma in bone in the museums of the London hospitals, and distinctly due to injury, but I select one example only —a striking one. A girl, aged sixteen, was bitten by a dog in the thigh three months before she came under observation. A periosteal sarcoma of bone developed, necessitating amputation of the thigh.

What, then, is the immediate connection between injury of the bone and the development of a new growth? Let us see what happens in injury of the bone in the ordinary way. An effusion of blood takes place either beneath the periosteum or into the cancellous tissue, and may follow one of three courses. The new cells either break down and form pus, or are absorbed and disappear, or become developed into fibrous tissue or new bone. But in sarcoma we may assume that the inflammatory cells of the effusion take a course which simulates exactly neither of the three processes mentioned above. They are not absorbed, they do not break down, nor are they built up into true fibrous tissue. They find their *rôle* in the formation of lowly-organised, feebly-vitalised cell material, which probably plays the part of tumour-germs. Clinically, the chief interest in this question of the formation of new growths of bone after injury rests in the diagnosis of such swellings from chronic inflammatory thickenings of the bone, and the distinction is often excessively difficult. In both an injury is frequently the starting-point, but in chronic ostitis we frequently find some of the symptoms of inflammation present, such as local heat, redness, swelling, and pain. Then, too, if we remember that the surface of a new growth is irregular, its outline is ill-defined and gradually merges into the surrounding tissues, the movements of the neighbouring joint within certain limits are free, enlargement is usually rapid and continuous, and the lymphatic glands are affected, we have much to aid us in clearing up the diagnosis. Mr. Holmes[1] however, alludes to a case under the care of Cæsar Hawkins, in which abscess and growth were combined. On incision pus and blood escaped, and subsequently uncontrollable arterial hæmorrhage ensued. After amputation a soft sarcoma was found to have been incised. It grew from the os calcis, and implicated the posterior tibial vessels. But I think that, except in such very rare cases as Mr. Holmes's, the use of the exploring needle is quite sufficient to clear up the diagnosis. In the case of sarcoma bright blood will be withdrawn, in the case of chronic ostitis probably pus or only a little serum.

With reference to the question of treatment, when malignant new growth on the surface of bone is diagnosed, only one course is possible, viz., amputation through the joint or bone above. Certain encapsuled forms of sarcoma, which grow in the interior and at the ends of the long bones, lend themselves to a less radical mode of treatment, viz., enucleation. This is applicable chiefly to sarcomata growing within the ends of the bones of the forearm. In other situations, on account of the great size of the growth, this proceeding is not possible, and amputation is the only resort.

[1] "System of Surgery," 2nd Edition, vol. ii., p. 317.

